# Determining intracellular and extracellular activities of azithromycin against azithromycin-resistant *Salmonella* Typhi and their association with clinical treatment responses

**DOI:** 10.1016/j.ebiom.2026.106447

**Published:** 2026-08-22

**Authors:** Ngo Ngoc Phuong Thuy, Voong Vinh Phat, Sabina Dongol, Nguyen Hoang Thu Trang, Sumit Bista, Quynh Nguyen, Chau Vinh, Guy E. Thwaites, Buddha Basnyat, Christopher M. Parry, Stephen Baker, Maia A. Rabaa, Jay C.D. Hinton, Abhilasha Karkey, Pham Thanh Duy

**Affiliations:** a Oxford University Clinical Research Unit, Ho Chi Minh City, Vietnam; b Oxford University Clinical Research Unit, Kathmandu, Nepal; c Centre for Tropical Medicine and Global Health, University of Oxford, Oxford, UK; d Department of Clinical Sciences, Liverpool School of Tropical Medicine, Liverpool, L3 5QA, UK; e A∗STAR Infectious Diseases Labs (A∗STAR IDL), Agency for Science, Technology and Research (A∗STAR), 8A Biomedical Grove, Immunos #05-13, Singapore, 138648, Singapore; f Institute of Infection, Veterinary and Ecological Sciences, University of Liverpool, Liverpool, UK

**Keywords:** Typhoid fever, *Salmonella* Typhi, Azithromycin resistant, THP-1 macrophages, Azithromycin

## Abstract

**Background:**

The spread of extensively drug-resistant (XDR) *Salmonella* Typhi variants in South Asia has severely limited treatment options for typhoid fever, a life-threatening systemic infection affecting millions. Azithromycin has become the last effective oral drug against XDR typhoid; however, despite increasing azithromycin resistance, limited data exists concerning the correlation between in vitro susceptibility and clinical treatment efficacy.

**Methods:**

We developed a THP-1 macrophage infection model to evaluate time- and concentration-dependent azithromycin activity against intracellular and extracellular *S.* Typhi, including azithromycin-susceptible (MIC ≤16 mg/L) and azithromycin-resistant isolates (MIC ≥ 32 mg/L). The accumulation of azithromycin in THP-1 macrophages was quantified using LC-MS/MS. Pharmacological modelling was used to estimate key parameters (E_max_, E_min_, C_s_, C_si_), and compare azithromycin efficacy between intracellular and extracellular compartments. When available, in vitro findings were correlated with clinical treatment responses.

**Findings:**

Azithromycin accumulated in THP-1 macrophages at concentrations 20–72 fold higher than in extracellular medium after 24 h. This intracellular accumulation was associated with concentration-dependent killing of *S.* Typhi in the intracellular assays, while only bacteriostatic effects were observed extracellularly. Importantly, pharmacological modelling data estimated that azithromycin concentrations ranging from 46.86 to 123.78 mg/L were sufficient to inhibit intracellular growth of azithromycin-susceptible isolates. In contrast, concentrations exceeding 460.63 mg/L were required to suppress intracellular growth of azithromycin-resistant isolates, substantially higher than the C_max_ typically observed in human white blood cells (114.0–146.0 mg/L) following a 3 day course of oral azithromycin. Notably, among those with available data, three patients infected with azithromycin-resistant *S.* Typhi failed to respond clinically to azithromycin treatment.

**Interpretation:**

We have established a THP-1 macrophage model to characterise the intracellular pharmacodynamics of azithromycin. Our findings indicate that azithromycin is likely ineffective for treating infections caused by azithromycin-resistant *S.* Typhi, warranting further clinical validation.

**Funding:**

Wellcome International Training Fellowship.

**Research in context**

**Evidence before this study**

“We searched PubMed and Web of Science using the terms: (“Typhoid fever” OR “enteric fever” OR “Salmonella Typhi” OR ″S. Typhi”) AND (“azithromycin-resistant” OR “azithromycin resistant” OR “azithromycin resistance” OR “macrolide resistance” OR “macrolide-resistant” OR “macrolide resistant” OR “azithromycin MIC”), NOT (“non-typhoidal” OR “nontyphoidal” OR “NTS”), limited to human studies published between January 1, 2005, and September 1, 2025. We included studies reporting phenotypic or genotypic azithromycin resistance or clinical outcomes, and excluded proceedings papers, non-typhoidal Salmonella studies, and studies without azithromycin MIC data. After screening titles, abstracts, and full texts and removing duplicates and review articles, 26 publications met the eligibility criteria. There was only one paper assessing bactericidal activity of azithromycin against *S.* Typhi. Emerging azithromycin-resistant *S.* Typhi (MIC >16 mg/L) has been reported in Asia, largely driven by independent acquisitions of *acrB* R717Q/R717L substitutions. However, the clinical efficacy of azithromycin against isolates with MICs >16 mg/L is unknown. Notably, no studies have evaluated intracellular and extracellular azithromycin activity against *S.* Typhi, highlighting key gaps in linking in vitro susceptibility with clinical efficacy of current azithromycin regimens against resistant strains.

**Added value of this study**

We established a THP-1 macrophage infection model to measure intracellular and extracellular activity of azithromycin against *S.* Typhi. Azithromycin accumulated within macrophages, corresponding to its concentration-dependent intracellular killing, while only exhibiting bacteriostatic effects extracellularly. Intracellular inhibition was achieved at clinically attainable intracellular concentrations for susceptible isolates, whereas resistant isolates required concentrations exceeding those typically observed in human white blood cells. Consistent with these findings, three patients infected with azithromycin-resistant *S.* Typhi failed azithromycin treatment. Together, these findings provide a mechanistic link between azithromycin resistance, diminished intracellular activity, and treatment failure.

**Implications of all the available evidence**

Our findings indicate that azithromycin is likely ineffective for treating infections caused by azithromycin-resistant *S.* Typhi, warranting further clinical studies to validate these findings.

## Introduction

Typhoid fever is a life-threatening systemic infection predominantly caused by *Salmonella enterica* serovar Typhi (*S.* Typhi). Despite significant progress in typhoid control worldwide, the disease remains a significant public health problem in many resource-limited settings. In the pre-antibiotic era, the mortality rate of typhoid fever was as high as 15%^1^; however, antimicrobial treatment has since reduced this to less than 1%.^2^ Historically, first-line drugs for the treatment of typhoid fever included chloramphenicol, ampicillin, and trimethoprim-sulfamethoxazole.^3^ The emergence and subsequent worldwide spread of multidrug-resistant and fluoroquinolone-resistant *S.* Typhi strains in the 1990s has led to the recommendation of alternative therapies by WHO, including ciprofloxacin, ceftriaxone, cefixime (3rd/4th-generation cephalosporins) and azithromycin (macrolide).^4^

In 2016, a large outbreak of extensively drug-resistant (XDR) *S.* Typhi with resistance to first-line drugs, fluoroquinolones, and third-generation cephalosporins was reported in Pakistan,^5^ followed by endemic circulation and regional spread.^6^ Consequently, azithromycin has become the only oral drug effective against XDR typhoid. Of particular concern, recent studies from Bangladesh, Pakistan, Nepal, Singapore, and India have reported *S.* Typhi isolates exhibiting in vitro resistance to azithromycin with MIC values ≥ 32 mg/L.^7^, ^8^, ^9^, ^10^, ^11^ These isolates have emerged through independent evolutionary events, each acquiring a similar point mutation at codon 717 in the *acrB* efflux pump gene.^7^^,^^8^^,^^11^

The emergence of azithromycin-resistant *S.* Typhi raises significant concerns about the efficacy of current azithromycin regimens, and poses an immediate threat of untreatable typhoid fever should such mutations arise in XDR isolates. The clinical breakpoints for azithromycin have been determined based on the MIC distribution of the wild-type *S.* Typhi population,^12^ and patients infected with isolates exhibiting azithromycin MICs ≤16 mg/L generally respond well to azithromycin.^13^ However, there is limited clinical data available for *S.* Typhi isolates with azithromycin MICs >16 mg/L, and thus the treatment efficacy of azithromycin among patients infected with these organisms is unknown.

Azithromycin is clinically effective for typhoid treatment due to its high tissue penetration, long half-life and sustained intracellular concentrations, making it suitable for intracellular pathogens like *S.* Typhi that thrive inside human cells.^14^ This drug can achieve concentrations >100 fold in human white blood cells compared to plasma.^15^^,^^16^ Despite its favourable pharmacokinetic properties, the activity of azithromycin against intracellular *S.* Typhi and the extent of correlation between in vitro susceptibility and in vivo efficacy was unknown. In this study, we established a THP-1 macrophage model to measure the intracellular and extracellular activity of azithromycin against *S.* Typhi and provide novel insights into their correlation with azithromycin treatment responses.

## Methods

### Bacterial strains and MIC determination

Sixteen *S.* Typhi isolates were used in this study, including 8 azithromycin-susceptible (Azith^S^) (MIC ≤16 mg/L) and 8 azithromycin-resistant (Azith^R^) isolates (MIC ≥32 mg/L) (Supplementary Table S1). Azith^S^ isolates were obtained from a randomised controlled trial conducted in Nepal comparing gatifloxacin versus ceftriaxone for treatment of enteric fever.^17^ Six Azith^R^ isolates were identified from routine surveillance at Patan Hospital, Nepal^7^ and two (ACT105-032, ACT105-033) from the completed ACT-South Asia trial, comparing azithromycin (20 mg/kg/day oral dose once daily) and cefixime (20–30 mg/kg/day oral dose in two divided doses for 7 days) versus azithromycin (20 mg/kg/day oral dose once daily for 7 days) and cefixime-matched placebo for 7 days, for the treatment of uncomplicated patients with typhoid (https://clinicaltrials.gov/ct2/show/NCT04349826).^18^ For the ACT-SA trial, a composite outcome of treatment failure is defined by either of the following events: 1. Clinical failure: persistence of fever on day 7 post treatment initiation OR The need for rescue treatment OR The development of any complication OR Syndromic enteric fever relapse within 28 days of initiation of treatment. 2. Microbiological failure: a positive blood-culture for *S.* Typhi or *S.* Paratyphi on day 7 of treatment regardless of the presence of fever OR blood culture-confirmed typhoid fever relapse within 28 days of initiation of treatment.^18^ The trial was conducted from May 2021 to December 2025, and a total of 2150 participants were enrolled.

The MICs of azithromycin against *S.* Typhi were determined by E-test (bioMérieux) in Mueller–Hinton agar, following the manufacturer's recommendations. *Staphylococcus aureus* ATCC 29213 was included for quality control in accordance with CLSI M100 (2020 edition), with the observed azithromycin MIC (1 mg/L) of *S. aureus* ATCC 29213 falling within the CLSI-approved quality control range.

Whole genome sequencing (WGS) was performed for five isolates using an Illumina Hiseq 2500, while publicly available genomic data was downloaded from ENA for the remaining isolates. WGS data were subjected to quality assessment by FastQC v0.11.5^19^ and genetic profiling by Typhi Mykrobe pipeline.^20^ Genetic profiles including genotype and the mutation in the *acrB* gene are shown in Supplementary Table S1.

### Cell culture

The intracellular activity of azithromycin against *S.* Typhi was assessed using a THP-1 macrophage model differentiated from a human acute monocytic leukaemia cell line (ATCC® TIB-202™, RRID: CVCL_0006).^21^ THP-1 macrophages closely replicate the function and biology of primary human macrophages,^22^ and have been widely used to evaluate the intracellular activity of antimicrobials against a wide range of organisms, including *S.* Typhi.^22^, ^23^, ^24^, ^25^, ^26^

For cell culture, human monocytic THP-1 cells (ATCC® TIB-202™, RRID: CVCL_0006) were grown in Roswell Park Memorial Institute medium (RPMI-1640) (Sigma, USA) supplemented with 10% FBS (Gibco, USA), 1 mM sodium pyruvate (Sigma, USA), 2 mM GlutaMAX (Thermofisher Scientific, USA), 10 mM HEPES buffer (Sigma, USA), 50 μg/mL penicillin and streptomycin (Sigma, USA) at 37 °C in a 5% CO_2_ incubator. Cells were routinely tested for mycoplasma contamination using the MycoAlert® Mycoplasma Detection Kit (Lonza, USA). THP-1 cells were seeded into a 96-well plate at a density of approximately 10^5^ cells per well and differentiated into macrophages by 48 h incubation with 100 ng/mL phorbol 12-myristate 13-acetate (PMA) (Sigma, USA), followed by 24 h incubation in medium without PMA.^22^

### THP-1 cell viability

THP-1 cells were seeded into a 96-well plate at a density of approximately 10^5^ cells per well and differentiated into macrophages as described above. Triton X-100 0.05% was used to induce maximal cell death. Propidium iodide (PI) (Sigma, USA) was diluted with water and added to each well to a final concentration of 50 μg/mL. All experiments included untreated control cells, 100% death cell control and blank wells. The FLUOstar OPTIMA microplate reader (BMG LABTECH) was set to fluorescent mode with an excitation wavelength of 540 nm and an emission wavelength of 610 nm. The PI uptake assay was used to assess the viability of THP-1 cells upon treatment with azithromycin concentrations ranging from 6 mg/L to 256 mg/L, or when infected with *S.* Typhi with selected multiplicity of infection (MOI) and time. All experiments were performed in three biological replicates.

### Accumulation of azithromycin inside THP-1 macrophages

A total of 10^5^ THP-1 cells were seeded in each well of a 96-well plate and differentiated into macrophages as described above. THP-1 macrophages were incubated at 37 °C in conditions containing 5% CO_2_ for up to 24 h with 0.4, 8, 16, and 128 μg/mL of azithromycin in RPMI-1640 with 10% FBS, 1 mM sodium pyruvate, 2 mM GlutaMAX and 10 mM HEPES buffer. After 30 min, 2 h, 4 h, 24 h of incubation, the cells were washed three times with PBS and lysed with 0.1% Triton X-100. The azithromycin concentrations in cell lysates were measured by the LC-MS/MS. Briefly, the LC-MS/MS system utilised a mobile phase consisting of acetonitrile–10 mM ammonium acetate buffer (35:65). Analytes were separated on a Zorbax C8 XDB column (50 × 4.6 mm; 3.5 μm) with roxithromycin used as the internal control. All experiments were performed in three biological replicates.

The intracellular concentration of azithromycin was calculated using the following equation,^27^ in which the average macrophage volume was 4989.9 μm^3^^28^:

**Total volume of cells** (μL) = number of cells in one well x average macrophage volume (μL)

**Intracellular azithromycin concentration** (mg/L) = Azithromycin concentration in lysate (mg/L) x lysate volume (μL) / Total volume of cells (μL)

Concentrations of azithromycin accumulation within THP-1 macrophage after 24 h were used to construct a non-linear model, depicting the relationship between intracellular and extracellular azithromycin concentrations. The data were fitted to a one-phase exponential association model according to the following equation:

$$Y=Ymax\times \left(1-e^{-kX}\right)$$

In this equation, Y represents the intracellular azithromycin concentration, and X represents the extracellular azithromycin concentration (mg/L). Y_max_ represents the maximal intracellular accumulation achieved at saturating extracellular concentrations, whereas k is the rate constant, reflecting the efficiency of intracellular drug accumulation as extracellular concentration increases.

### Concentration- and time-dependent killing assays

For intracellular activity of azithromycin against *S.* Typhi, ∼10^5^ THP-1 cells were seeded in each well of a 96-well plate and differentiated as described above. THP-1 macrophages were infected with *S.* Typhi at an MOI of 1, 10 and 100, for durations of 15, 30, 45, 60, 90, and 120 min.

After infection, non-phagocytosed *S.* Typhi was removed by washing three times with PBS, followed by incubation with RPMI-1640 containing 100 μg/mL gentamicin for 30 min. The infected THP-1 macrophages were exposed to media containing different concentrations of azithromycin, ranging from 0.1 × MIC to 8 × MIC for Azith^S^ strains and from 0.4 to 128 mg/L for Azith^R^ strains. Cells were subsequently incubated at 37 °C in condition containing 5% CO_2_ without shaking.

Intracellular bacteria were enumerated after 0.5, 2, 4 and 24 h of incubation. After incubation, media was removed and the cell layer was washed twice with PBS. THP-1 macrophages were lysed by adding 0.05% Triton X-100 to collect the intracellular bacteria. Cell lysates were serially diluted, plated on LB agar and incubated overnight at 37 °C to determine the colony forming units (CFUs).

### Extracellular activity of azithromycin against *S.* Typhi

The extracellular killing activity of azithromycin against *S.* Typhi was determined in the same culture medium of THP-1 macrophage (RPMI-1640 containing 10% FBS, 1 mM sodium pyruvate, 2 mM GlutaMAX, 10 mM HEPES buffer) in the presence of different concentrations of azithromycin, consistent with the intracellular concentration- and time-dependent killing assays. The numbers of CFUs were also enumerated after 0.5, 2, 4 and 24 h of incubation.

### Data analysis

Data analyses were done with GraphPad Prism version 10.2.0 (GraphPad Prism, USA). The killing data from all intracellular and extracellular experiments were analysed by fitting a sigmoidal dose–response curve (Hill equation) with Hill slope = 1 (three-parameter logistic equation).^29^

$$Y=Emin+\frac{Emax-Emin}{10^{-logEC50+X}+1}$$

The following pharmacological parameters were estimated from each concentration–response curve: minimal relative efficacy (E_min_) in log_10_ CFU/mL, corresponding to the increase in CFU for an infinitely low antibiotic concentration compared to the initial inoculum; maximal relative efficacy (E_max_) in log_10_ CFU/mL, corresponding to the decrease in CFU for an infinitely high antibiotic concentration compared to the initial inoculum; relative potency (EC_50_) in mg/L or multiples of MIC, corresponding to the antibiotic concentration yielding a value of CFU halfway between E_min_ and E_max_; static concentration (C_s_) in mg/L or multiples of MIC, corresponding to the antibiotic concentration at which there is no apparent change in CFU compared to the initial inoculum.

### Statistics

Statistical analyses were performed using GraphPad Prism version 10.2.0 (GraphPad Prism, USA). Dose–response killing curves were fitted using the Hill equation as described above. The fitted parameters included the maximum effect (E_max_) and the minimum effect (E_min_). Parameter estimates were reported together with 95% confidence intervals (95% CI). The goodness-of-fit of each model was evaluated using the coefficient of determination (R^2^). For the PI uptake assay of THP-1 cell viability following azithromycin treatment or *S.* Typhi infection, fluorescence levels between groups were compared using the Kruskal–Wallis test. Differences were considered to be statistically significant at P-value <0.05.

### Ethics

The studies from which the isolates and corresponding clinical data were derived for this analysis have been approved by the Ethics Committee of the Nepal Health Research Council and the Oxford Tropical Research Ethics Committee (UK) (reference number 28–20). This study was conducted in accordance with the principles of the 2024 Declaration of Helsinki. Written informed consent was obtained from all participants prior to sample and data collection.

### Role of funders

The funders had no role in the design and conduct of the study; collection, management, analysis, and interpretation of the data; writing, review, or approval of the manuscript; and decision to submit the manuscript for publication.

## Results

### Establishment of THP-1 macrophage model for measuring intracellular killing activity of azithromycin against *S.* Typhi

**Fig. 1:**
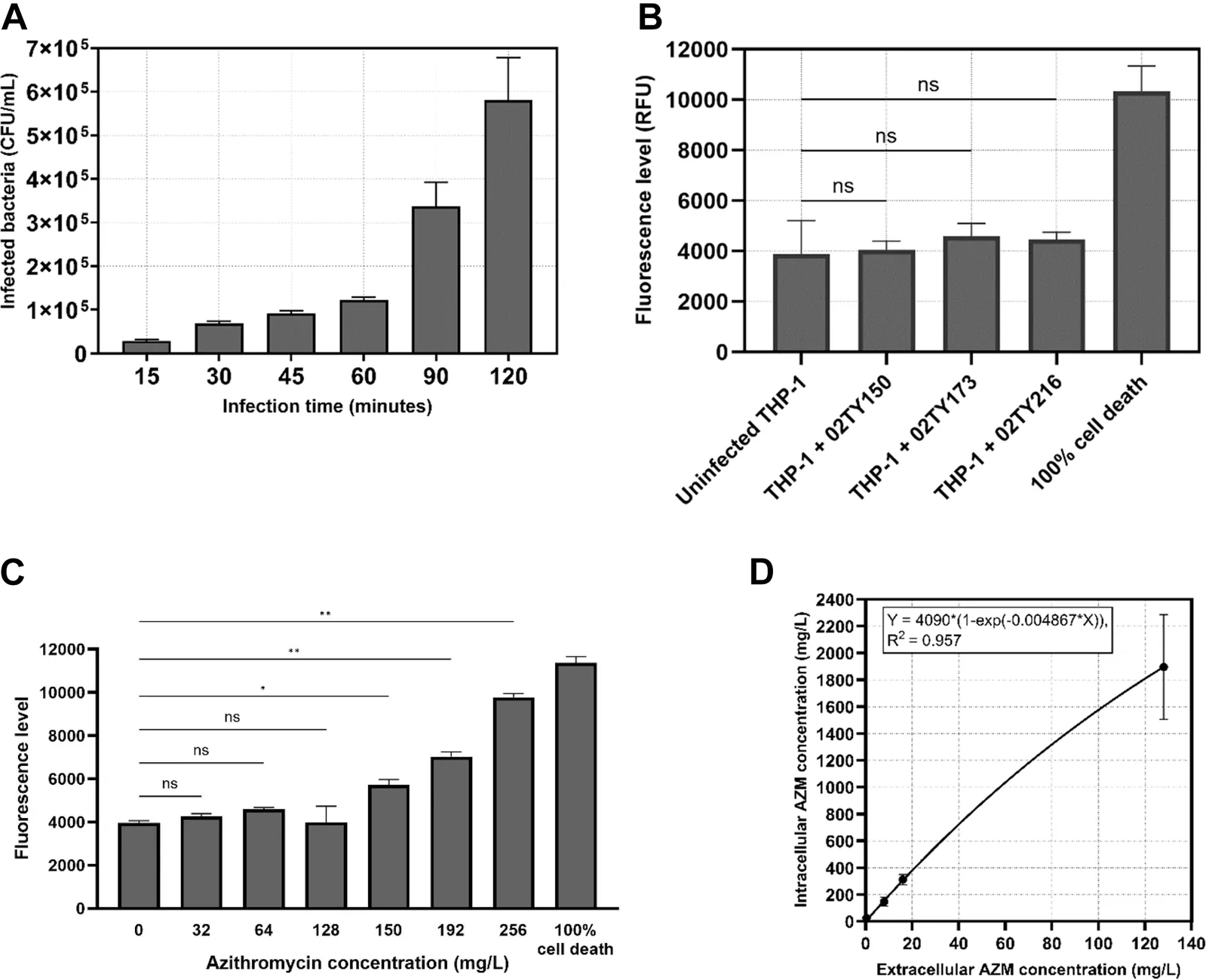
Optimisation of the THP-1 macrophage model to assess intracellular activity of azithromycin against *S.* Typhi. **A.** The infection duration of *S.* Typhi in THP-1 macrophages. THP-1 macrophages were infected with *S.* Typhi (MOI = 10) for the indicated durations and intracellular bacteria were quantified by CFU enumeration. After 45 min of infection, intracellular *S.* Typhi reached ∼10^5^ CFU/mL, consistent with the bacterial concentration used in the extracellular experiments; this infection duration was used for subsequent experiments. The experiments were performed in triplicate **B.** THP-1 cell viability upon *S.* Typhi infection with MOI of 10 and infection time of 45 min. Propidium iodide (PI) uptake assay was used to assess cell viability, where higher fluorescence level indicates lower cell viability. Uninfected THP-1 macrophages were used as negative control, and killed THP-1 cells as positive control (100% cell death). Three biological replicates were performed for each condition **C.** THP-1 cell viability after 24 h exposure to azithromycin at different concentrations. PI uptake assay was used to assess cell viability, where higher fluorescence level indicates lower cell viability. Unexposed THP-1 macrophages were used as negative control, and killed THP-1 cells as positive control (100% cell death). Three biological replicates were performed for each condition. **D.** The accumulation of azithromycin into THP-1 macrophage after 24 h incubation. Initial extracellular concentration (x-axis) and the intracellular concentration of azithromycin (y-axis). The non linear curve was fitted to the data using GraphPad Prism software with the equation presents on the graph. Data are expressed as mean values (n = 3). Error bars represent SD. Kruskal–Wallis test was used for statistical analysis in Figure B and C, with the P-values indicated as follows: ns, no significant; ∗P ≤ 0.05; ∗∗P ≤ 0.01. Detailed statistical results are provided in Supplementary Tables S3 and S4.

Following the evaluation of MOIs, infection durations and cell viability, an MOI of 10 and infection duration of 45 min were selected. These experimental conditions resulted in an intracellular concentration of ∼10^5^ CFU/mL of *S.* Typhi, which was consistent with the concentration used in the extracellular experiments (Fig. 1A), without affecting the cell viability (Fig. 1B). Our data showed that extracellular azithromycin concentrations exceeding 150 mg/L were toxic to the cells; therefore, a maximum testable concentration of azithromycin was determined to be 128 mg/L (Fig. 1C). The accumulation of azithromycin in THP-1 macrophages increased with time and concentration (Fig. 1D, Table 1). At an initial extracellular concentration of 0.4 mg/L, azithromycin was undetectable within the THP-1 macrophages after 30 min. However, after 24 h, intracellular accumulation reached 26.61 mg/L (±8.56), which was 72-fold higher than the extracellular concentration. At the extracellular concentrations of 8, 16, and 128 mg/L, the intracellular azithromycin concentrations were approximately 20-fold higher after 24 h of incubation (Table 1).

**Table 1:** Intracellular concentration of azithromycin (mg/L) over 24 h (means ± SDs, n = 3) and intracellular-to-extracellular (I/E) concentration ratio at 24 h according to corresponding extracellular azithromycin concentration.

Extracellular concentration	0.5 h	2 h	4 h	24 h	I/E ratio
0.4 mg/L	0	1.03 ± 1.79	2.18 ± 3.77	26.61 ± 8.56	72.61 ± 32.02
8 mg/L	35.77 ± 0.95	84.85 ± 71.24	63.28 ± 10.55	146.15 ± 33.93	19.56 ± 4.97
16 mg/L	109.38 ± 7.37	166.01 ± 50.79	172.20 ± 6.74	311.38 ± 39.24	20.60 ± 2.83
128 mg/L	966.54 ± 85.72	999.78 ± 86.04	1233.33 ± 52.60	1896.32 ± 389.34	19.22 ± 4.34

### Azithromycin activities against intracellular and extracellular *S.* Typhi isolates

For the Azith^S^ *S.* Typhi isolates, azithromycin exhibited a concentration-dependent killing effect in the intracellular assays, with increased bacterial clearance observed at higher drug concentrations. Specifically, azithromycin concentrations at 8 × MIC (32–128 mg/L) resulted in a 0.6–1.63 log_10_ CFU/mL reduction in intracellular bacterial load after 24 h (Supplementary Figure S1). Conversely, azithromycin showed a typical bacteriostatic effect in the extracellular experiments, with no significant difference in inhibitory activity across concentrations ranging from 1× to 8 × MIC (Supplementary Figure S1).

**Fig. 2:**
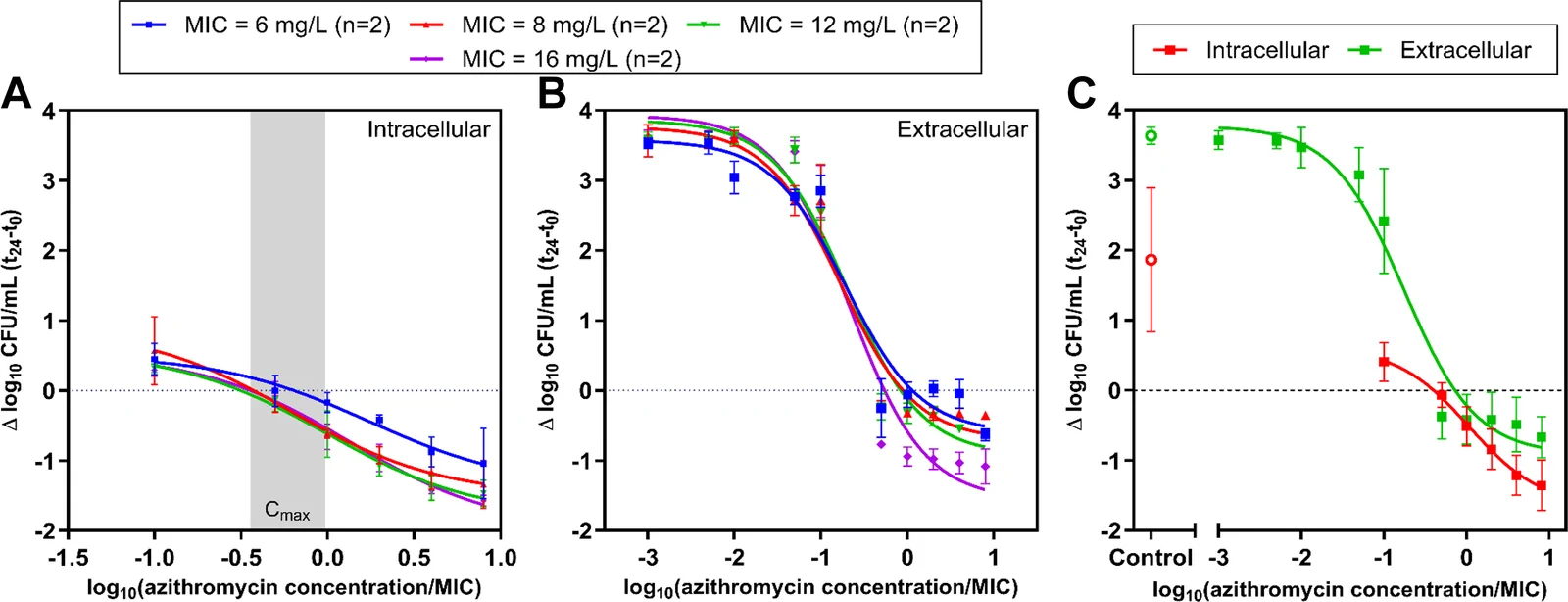
Concentration-dependent activities of azithromycin against intracellular and extracellular Azith^S^*S.* Typhi isolates. Left panel (**A**) and middle panel (**B**): The y-axis shows the change in the number of CFU per mL (Δ log_10_ CFU; means ± SD; with three biological replicates per isolate) from the initial inoculum after 24 h of incubation, the x-axis shows as log_10_ of extracellular azithromycin concentrations standardised as multiples of MIC of the azithromycin-susceptible isolates. Right panel (**C**): Concentration-dependent activities of azithromycin against all intracellular (red line) and extracellular (green line) azithromycin-susceptible isolates. Open circles show the change in the number of CFU per mL from the initial inoculum after 24 h of incubation in control conditions (without antibiotic). The grey zone in the left panel shows the estimated extracellular concentration of azithromycin, corresponding to the maximal concentration of azithromycin in human white blood cells on day 1 and day 3 of a 3 day oral 500 mg azithromycin regimen (C_max_ = 42.9 mg/L on day 1 and C_max_ = 114 mg/L on day 3).^30^

The time-killing data of Azith^S^ isolates, representing four MIC groups, were analysed using a sigmoidal function to model the relationship between azithromycin concentration and bacterial reduction over time (Fig. 2). The estimated maximum efficacy (E_max_) of azithromycin ranged from a 1.39 to 1.99 log_10_ CFU/mL reduction against intracellular Azith^S^ isolates, and from a 0.61 to 1.56 log_10_ CFU/mL reduction in extracellular conditions, indicating greater killing activity in the intracellular model (Table 2). Consistent with enhanced intracellular activity, azithromycin showed lower E_min_ values, representing residual bacterial burden, against intracellular isolates (0.51–0.91 log_10_ CFU/mL) compared to extracellular isolates (3.58–3.93 log_10_ CFU/mL) (Table 2). Notably, the intracellular static concentration (C_si_), defined as the azithromycin concentration resulting in no apparent bacterial growth compared to the initial inoculum, was estimated to range from 46.86 to 123.78 mg/L, corresponding to 6.26–12.69-fold the MIC (Table 2). In contrast, the static concentration (C_s_) in extracellular experiments was close to MIC values, ranging from 0.54 to 1.13-fold of the MIC (Table 2). An additional difference was observed at the 0.1 × MIC concentration (0.6–1.6 mg/L), where azithromycin markedly inhibited the intracellular growth of *S.* Typhi. However, at this concentration, azithromycin exerted a minimal effect on extracellular growth of *S.* Typhi (Fig. 2C). In general, the activity of azithromycin was consistent across isolates with varying MIC values, as data plotted as a sigmoidal function of multiples of their MICs resulted in nearly indistinguishable concentration-dependent curves.

**Table 2:** Pharmacological parameters estimated from dose–response sigmoidal model of azithromycin against Azith^S^*S.* Typhi isolates with different MIC groups.

*S.* Typhi isolates	E_min_ (95% CI)^a^	E_max_ (95% CI)^b^	EC_50_^c^ (×MIC)	EC_50i_^d^ (mg/L)	C_s_^e^ (×MIC)	C_si_^f^ (mg/L)	R^2^	Adjusted R^2^
Intracellular								
MIC = 6 mg/L (n = 2)	0.51 (0.26–0.77)	−1.39 (−1.81 to −0.97)	1.74	206.94	0.64	76.15	0.803	0.791
MIC = 8 mg/L (n = 2)	0.91 (0.54–1.29)	−1.50 (−1.74 to −1.26)	0.62	98.59	0.37	59.22	0.869	0.861
MIC = 12 mg/L (n = 2)	0.57 (0.35–0.80)	−1.80 (−2.02 to −1.58)	0.99	235.91	0.31	75.17	0.923	0.919
MIC = 16 mg/L (n = 2)	0.53 (0.36–0.70)	−1.99 (−2.02 to −1.78)	1.35	428.56	0.36	113.59	0.953	0.951
Azith^S^*S.* Typhi isolates (n = 8)	0.62 (0.46–0.78)	−1.65 (−1.81 to −1.49)	1.06	126.63–337.69	0.39	46.86–123.78	0.832	0.829
Extracellular								
MIC = 6 mg/L (n = 2)	3.58 (3.36–3.79)	−0.61 (−0.84 to −0.39)	0.19	NA	1.13	NA	0.936	0.934
MIC = 8 mg/L (n = 2)	3.76 (3.58–3.95)	−0.71 (−0.90 to −0.53)	0.17	NA	0.88	NA	0.958	0.957
MIC = 12 mg/L (n = 2)	3.86 (3.69–4.03)	−0.93 (−1.11 to −0.75)	0.20	NA	0.82	NA	0.968	0.967
MIC = 16 mg/L (n = 2)	3.93 (3.71–4.15)	−1.56 (−1.82 to −1.32)	0.22	NA	0.54	NA	0.956	0.955
Azith^S^*S.* Typhi isolates (n = 8)	3.78 (3.66–3.90)	−0.91 (−1.04 to −0.79)	0.18	NA	0.72	NA	0.939	0.938

Parameters are derived from concentration–response curves shown in Fig. 2 using nonlinear regression based on the Hill equation.

NA: Not applicable.

a Relative minimal efficacy (log_10_ CFU/mL, 95% confidence interval): The increase in colony-forming units (CFU) for an infinitely low antibiotic concentration compared to the initial inoculum.

b Relative maximal efficacy (log_10_ CFU/mL, 95% confidence interval): The decrease in CFU for an infinitely high antibiotic concentration compared to the initial inoculum.

c Extracellular concentration of azithromycin (multiples of MIC) causing a reduction halfway between E_min_ and E_max_, as obtained from the Hill equation (with slope factor of 1).

d Intracellular concentration of azithromycin (mg/L) causing a reduction halfway between E_min_ and E_max_, as calculated from^c^ and the equation of the non-linear curve showing the accumulation of azithromycin into THP-1 macrophage after 24 h incubation (Fig. 1D).

e Extracellular concentration of azithromycin (multiples of MIC) resulting in no apparent bacterial growth compared to the initial inoculum, as determined by graphical interpolation.

f Intracellular concentration of azithromycin (mg/L) resulting in no apparent bacterial growth compared to the initial inoculum, as calculated from^e^ and equation of the non-linear curve showing the accumulation of azithromycin into THP-1 macrophage after 24 h incubation (Fig. 1D).

For the Azith^R^ *S.* Typhi isolates, although azithromycin also showed a concentration-dependent effect in the intracellular assays, its killing activity was only prominent at the highest concentration of 128 mg/L, with a 0.941–2.097 log_10_ CFU/mL reduction after 24 h of growth. In contrast, azithromycin also exhibited a bacteriostatic effect against extracellular Azith^R^ *S.* Typhi isolates at concentrations ≥8 mg/L depending on the MIC of each isolate (Supplementary Figure S2).

**Fig. 3:**
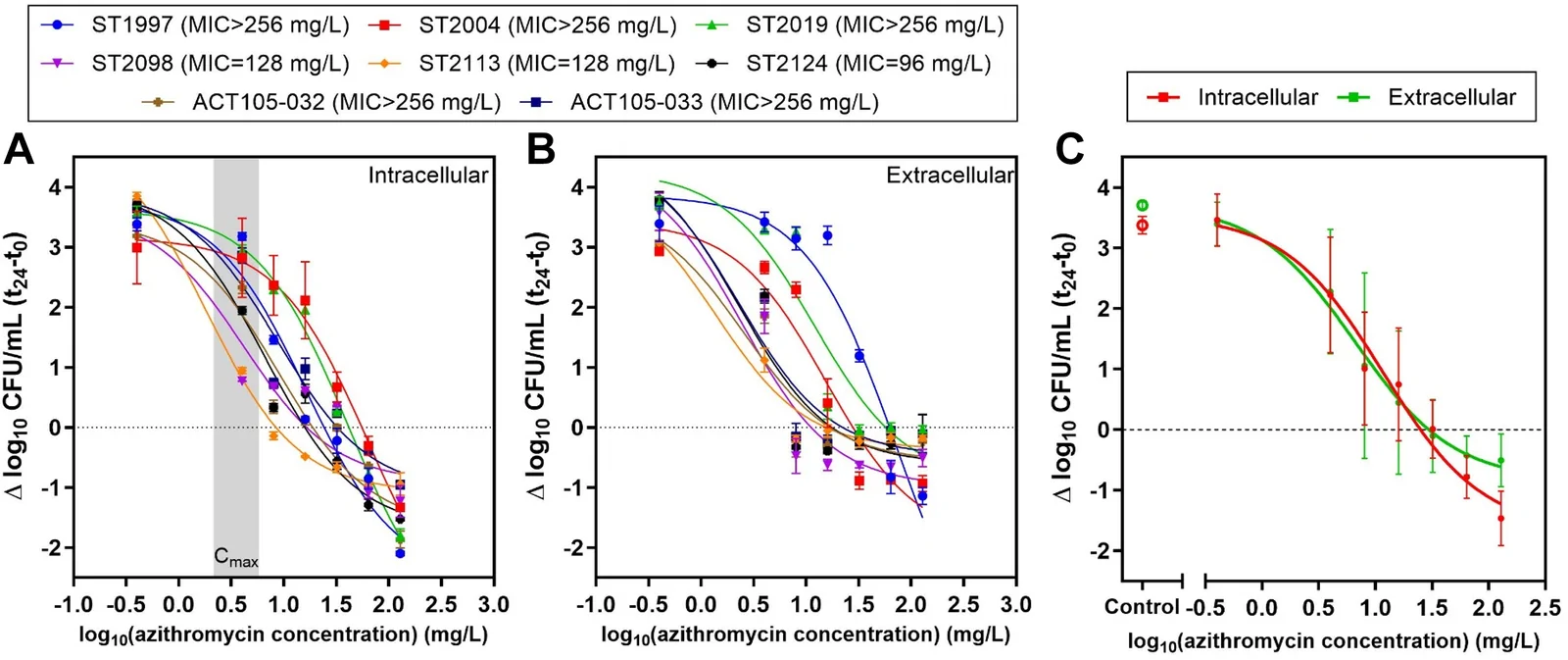
Concentration-dependent activities of azithromycin against intracellular and extracellular Azith^R^*S.* Typhi isolates. Left panel (**A**) and middle panel (**B**): The y-axis shows the change in the number of CFU per mL (Δ log_10_ CFU; means ± SD; with three biological replicates) from the initial inoculum after 24 h of incubation, the x-axis shows as log_10_ of extracellular azithromycin concentrations. The goodness of fit (R^2^) of each curve is shown on the graph. Right panel (**C**): Concentration-dependent activities of azithromycin against all intracellular (red line) and extracellular (green line) azithromycin-resistant isolates. Open circles show the change in the number of CFU per mL from the initial inoculum after 24 h of incubation in control conditions (without antibiotic). The grey zone in the left panel shows the estimated extracellular concentration of azithromycin corresponding to the maximal concentration of azithromycin in human white blood cells on day 1 and day 3 of a 3 day oral 500 mg azithromycin regimen (C_max_ = 42.9 mg/L on day 1 and C_max_ = 114 mg/L on day 3).^30^

The time-killing data of Azith^R^ isolates were also analysed using a sigmoidal function (Fig. 3).

The estimated E_max_ values ranged from a 0.91 to 3.67 log_10_ CFU/mL reduction against intracellular Azith^R^ isolates, and from a 0.37 to 4.62 log_10_ CFU/mL reduction in extracellular conditions (Table 3). Notably, while low concentrations of azithromycin (0.6–1.6 mg/L) effectively inhibited the intracellular growth of Azith^S^ isolates, a concentration of 4 mg/L failed to inhibit intracellular growth of Azith^R^ *S.* Typhi isolates (Fig. 3A). Importantly, for Azith^R^ *S.* Typhi isolates with MIC values > 256 mg/L, a minimum azithromycin concentration of 64 mg/L was required to inhibit intracellular growth (Fig. 3A). Achieving a bacteriostatic effect at this MIC level required intracellular static concentrations (C_si_) of azithromycin to exceed 356.9 mg/L. In contrast, for isolates with MIC value ranging from 96 to 128 mg/L, a static effect was achieved at concentrations of <300 mg/L (Table 3). In extracellular experiments, the static concentration (C_s_) of azithromycin for Azith^R^ isolates ranged from 11.14 to 62.46 mg/L, remaining below their respective MIC values (Table 3). In comparison, the C_s_ of azithromycin for Azith^S^ isolates were close to their MICs (Supplementary Table S2).

**Table 3:** Pharmacological parameters estimated from dose–response sigmoidal model of azithromycin against Azith^R^*S.* Typhi isolates.

*S.* Typhi isolates	MIC (mg/L)	E_min_ (95% CI)^a^	E_max_ (95% CI)^b^	EC_50_^c^ (mg/L)	EC_50i_^d^ (mg/L)	C_s_^e^(mg/L)	C_si_^f^ (mg/L)	R^2^	Adjusted R^2^
Intracellular									
ST1997	>256	3.76 (3.23–4.33)	−2.53 (−3.36 to −1.88)	16.25	311.01	23.99	450.72	0.950	0.945
ST2004	>256	3.16 (2.52–3.86)	−3.67 (−5.00 to −0.23)	64.28	1098.74	54.95	959.78	0.836	0.818
ST2019	>256	3.62 (3.40–3.82)	−3.58 (−4.34 to −2.97)	41.15	742.32	41.69	751.11	0.988	0.986
ST2098	128	3.59 (2.60–4.82)	−0.91 (−1.65 to −0.36)	4.186	82.48	16.60	317.44	0.865	0.850
ST2113	128	4.93 (4.56–5.00)	−1.08 (−1.21 to −0.94)	1.839	36.44	8.32	162.31	0.990	0.989
ST2124	96	3.99 (3.45–4.57)	−1.69 (−2.13 to −1.29)	6.693	131.08	15.69	300.70	0.960	0.955
ACT105-032	>256	3.44 (2.74–4.19)	−1.66 (−2.40 to −1.07)	9.072	176.66	18.76	356.90	0.911	0.901
ACT105-033	>256	3.93 (3.32–4.57)	−1.06 (−1.66 to −0.56)	8.740	170.33	31.94	588.85	0.927	0.919
Azith^R^*S.* Typhi isolates (n = 8)		3.54 (3.23–3.85)	−1.66 (−1.98 to −1.34)	11.71	226.58	24.55	460.63	0.825	0.822
Extracellular									
ST1997	>256	3.86 (3.40–4.34)	−4.62 (−8.78 to −2.67)	74.39	NA	62.46	NA	0.928	0.920
ST2004	>256	3.43 (2.87–4.04)	−1.90 (−2.70 to −1.25)	15.42	NA	27.54	NA	0.915	0.905
ST2019	>256	4.27 (3.38–5.00)	−0.90 (−1.83 to −0.14)	12.05	NA	57.25	NA	0.855	0.839
ST2098	128	4.43 (3.39–5.00)	−0.98 (−1.41 to −0.57)	2.45	NA	11.14	NA	0.903	0.892
ST2113	128	5.00 (3.13–5.00)	−0.37 (−0.58 to −0.16)	1.38	NA	15.14	NA	0.952	0.946
ST2124	96	4.65 (3.45–5.00)	−0.61 (−1.08 to −0.16)	2.29	NA	17.38	NA	0.876	0.862
ACT105-032	>256	3.70 (2.89–4.76)	−0.56 (−0.97 to −0.19)	2.56	NA	16.67	NA	0.872	0.858
ACT105-033	>256	4.60 (3.67–5.88)	−0.47 (−0.91 to −0.07)	2.29	NA	21.66	NA	0.926	0.874
Azith^R^*S.* Typhi isolates (n = 8)		3.73 (3.30–4.16)	−0.84 (−1.15 to −0.52)	6.64	NA	29.31	NA	0.704	0.703

Parameters are derived from concentration–response curves shown in Fig. 3, using nonlinear regression based on the Hill equation.

NA: Not applicable.

a Minimal relative efficacy (log_10_ CFU/mL, 95% confidence interval): The increase in colony-forming units (CFU) for an infinitely low antibiotic concentration compared to the initial inoculum.

b Maximal relative efficacy (log_10_ CFU/mL, 95% confidence interval): The decrease in CFU for an infinitely high antibiotic concentration compared to the initial inoculum.

c Extracellular concentration of azithromycin (mg/L) causing a reduction halfway between E_min_ and E_max_, as obtained from the Hill equation (with slope factor of 1).

d Intracellular concentration of azithromycin (mg/L) causing a reduction halfway between E_min_ and E_max_, as calculated from^c^ and equation of the non-linear curve showing the accumulation of azithromycin into THP-1 macrophage after 24-h incubation (Fig. 1D).

e Extracellular concentration of azithromycin (mg/L) resulting in no apparent bacterial growth compared to the initial inoculum, as determined by graphical interpolation.

f Intracellular concentration of azithromycin (mg/L) resulting in no apparent bacterial growth compared to the initial inoculum, as calculated from^e^ and equation of the non-linear curve showing the accumulation of azithromycin into THP-1 macrophage after 24-h incubation (Fig. 1D).

### Pharmacological comparison of azithromycin against intracellular *S.* Typhi isolates

**Fig. 4:**
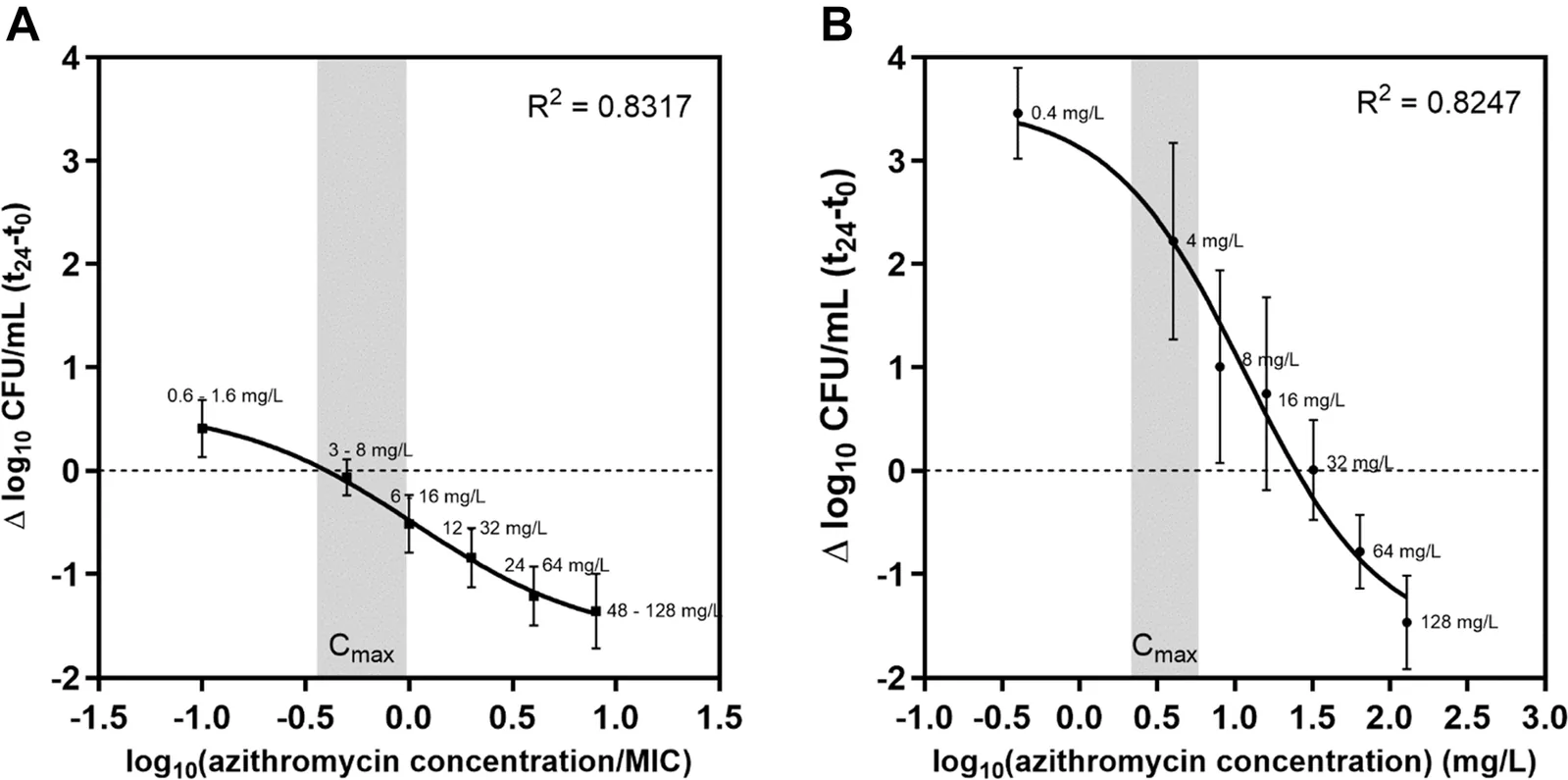
Concentration-response curve of intracellular *S.* Typhi after 24 h growth in azithromycin, comparing azithromycin-susceptible (**A**) and azithromycin-resistant isolates (**B**) The y-axis shows the change in the number of CFU per mL (Δ log_10_ CFU; means ± SD; 8 experimental isolates, each with three biological replicates) from the initial inoculum after 24 h of incubation. **A.** the x-axis shows as log_10_ of extracellular azithromycin concentrations standardised as multiples of MIC of the azithromycin-susceptible isolates, with the numbers above the curve showing the experimental concentrations relative to the isolate's MIC. **B.** the x-axis shows as log_10_ of extracellular azithromycin concentrations, with the numbers above the curve showing the tested concentrations for each azithromycin resistant isolate. The goodness of fit (R^2^) of each curve is shown on the graph. The grey zone shows the estimated extracellular concentration of azithromycin corresponding to the maximal concentration of azithromycin in human white blood cells on day 1 and day 3 of a 3 day oral 500 mg azithromycin regimen (C_max_ = 42.9 mg/L on day 1 and C_max_ = 114 mg/L on day 3).^30^

The intracellular time- and dose-dependent killing effects of azithromycin against all Azith^S^ and Azith^R^ isolates were combined and modelled using concentration–response curves fitted to the Hill equation (Fig. 4).

For Azith^S^ *S.* Typhi, our sigmoidal model demonstrated that azithromycin concentrations ranging from 2.36 to 6.31 mg/L were sufficient to inhibit intracellular bacterial growth (Fig. 4A). These concentrations corresponded to intracellular azithromycin levels of 46.86–123.78 mg/L, as estimated from our azithromycin accumulation model in THP-1 macrophages (Fig. 1D). Notably, these intracellular levels overlap with peak azithromycin concentrations (C_max_) observed in healthy human white blood cells, which reach 42.9 mg/L on day 1 and can increase to 114 mg/L by day 3 following a daily 500 mg oral dose. In contrast, for Azith^R^ *S.* Typhi isolates, our sigmoidal model estimated that the lowest effective extracellular concentration of azithromycin required to inhibit intracellular growth was 24.55 mg/L (Fig. 4B), corresponding to an intracellular concentration of 460.63 mg/L. This required intracellular level substantially exceeds the C_max_ observed in human white blood cells.

### Azithromycin treatment outcomes in azithromycin resistant typhoid cases

Among the two Azith^R^ typhoid cases (ACT105-032, ACT105-033) enrolled in the ACT-South Asia trial on 20th August 2023, neither responded to either azithromycin monotherapy or azithromycin-cefixime combination, necessitating rescue therapy with ceftriaxone. Specifically, the first patient was a 25-year-old, 64.2 kg man who presented with a 7 day history of fever (temperature 39.8 °C), headache, vomiting, and dry cough. Blood tests showed WCC 3.3 × 10^3^/μL, AST 254 U/L, and CRP 181 mg/L. Blood culture was positive for azithromycin-resistant *S.* Typhi. The isolate was ciprofloxacin-intermediate and susceptible to ceftriaxone, cefixime, chloramphenicol, ampicillin, and cotrimoxazole. The patient was treated with azithromycin 1 g once daily and cefixime 20 mg/kg/day in two divided doses for 7 days. The patient remained febrile and unwell at the end of therapy. A repeat blood culture performed at day 7 was again positive for *S.* Typhi, and rescue treatment with ceftriaxone was administered for a further 7 days. The final fever clearance time was 281 h (11.7 days). The second patient was a 20-year-old, 56 kg man who presented with an 8 day history of fever (temperature 39.6 °C), headache, diarrhoea, and dry cough. Blood tests showed WCC 3.7 × 10^3^/μL, AST 597 U/L, and CRP 191 mg/L. Blood culture was positive for azithromycin-resistant *S.* Typhi. The isolate was ciprofloxacin-intermediate and susceptible to ceftriaxone, cefixime, chloramphenicol, ampicillin, and cotrimoxazole. The patient was treated with azithromycin 1 g once daily for 7 days but remained febrile and unwell at the end of therapy. Repeat blood culture at day 7 was negative. The patient received rescue treatment with ceftriaxone for a further 7 days. The final fever clearance time was 425 h (17.7 days).

Furthermore, among the three further patients infected with Azith^R^ *S.* Typhi (ST1997, ST2004, ST2019) identified in our previous study, the one with available outcome failed to respond to a daily azithromycin dose of 1000 mg, requiring subsequent treatment with ceftriaxone.

## Discussion

Typhoid fever can lead to death or serious complications without appropriate antimicrobial treatment. Although typhoid conjugate vaccines have been pre-qualified by WHO and successfully implemented in several countries,^31^ effective antimicrobials remain essential to manage patients with typhoid fever. The spread of XDR typhoid^5^^,^^6^ and recent reports of carbapenem-resistant typhoid in Pakistan^32^ are concerning, as this means more azithromycin will likely be prescribed for typhoid management in highly endemic populations. Concerningly, the development of a single azithromycin resistance mutation in XDR *S.* Typhi strains can lead to untreatable typhoid. There is a significant gap in determining the efficacy of existing azithromycin doses for the treatment of azithromycin-resistant typhoid cases. A previous study has shown that three outpatients infected with Azith^R^ isolates did not respond to oral azithromycin^7^; furthermore, azithromycin treatment failure was occasionally observed in patients infected with Azith^S^ isolates (MIC ≤ 16 mg/L).^33^ Nonetheless, available clinical data are very limited, drawn from a small number of patients. As azithromycin can accumulate at high concentrations intracellularly, it is currently uncertain of how well in vitro azithromycin susceptibility testing correlates with in vivo efficacy. Furthermore, there has been only one intracellular study investigating azithromycin against *S.* Typhi, which was restricted to a single isolate, two concentrations, and did not measure intracellular drug levels.^34^ Here, we assessed the relationship between extracellular and intracellular azithromycin concentrations and their corresponding killing activities, enabling direct comparison with clinically relevant drug exposures and treatment responses. Therefore, this work provides mechanistic insight into the intracellular pharmacodynamics of azithromycin against *S.* Typhi and integrates our findings with clinical outcomes.

Our study demonstrates that the intracellular activity of azithromycin against *S.* Typhi is a more reliable predictor of treatment outcomes than traditional azithromycin susceptibility testing. This difference is attributed to the accumulation of azithromycin in THP-1 macrophages at concentrations 20–72 fold higher than extracellular concentrations, consistent with previous PK/PD studies in human volunteers.^15^^,^^30^ The high level of intracellular accumulation of azithromycin corresponded with its dose-dependent killing activity against intracellular *S.* Typhi, while only demonstrating bacteriostatic effect extracellularly. Through a series of concentration- and time-dependent killing assays, we successfully established pharmacological models to characterise azithromycin activity against intracellular and extracellular Azith^S^ and Azith^R^ isolates. These models enabled us to define critical concentration thresholds required to inhibit intracellular bacterial growth. Strikingly, our modelling data estimated that azithromycin concentrations ranging from 46.86 to 123.78 mg/L were sufficient to inhibit intracellular growth of Azith^S^ isolates, whereas concentrations exceeding 460.63 mg/L were required to suppress intracellular growth of Azith^R^ isolates. It is essential to note that the peak serum concentration (C_max_) of azithromycin typically ranges from 0.41 to 0.46 mg/L, while its concentration in white blood cells only reached 42.9 mg/L on day 1 and increased to as high as 114 mg/L on day 3, following oral human challenge with a daily regimen of 500 mg.^15^^,^^30^ Moreover, with a regimen of 2000 mg per day, azithromycin concentrations can reach up to 146 mg/L inside white blood cells,^30^ a concentration sufficient to inhibit intracellular growth of Azith^S^ isolates but remains far below the 460.63 mg/L required to inhibit the intracellular growth of Azith^R^ isolates. Previous PK simulations from a treatment trial, comparing azithromycin with ciprofloxacin in a controlled human infection model showed that a 500 mg daily regimen or a 1000 mg loading dose followed by 500 mg daily, increased intracellular azithromycin concentrations above the MIC within 24 h, while extracellular levels remained below the MIC; however intracellular concentrations did not exceed 100 mg/L. Additional simulations of alternative regimens (1000 mg daily or 500 mg twice daily) similarly showed that intracellular concentrations did not exceed 200 mg/L.^35^ These levels remain below the intracellular threshold required to effectively kill azithromycin-resistant strains.

Our findings suggest that the current dosing regimens of azithromycin, while effective against Azith^S^ *S.* Typhi with MICs <32 mg/L, will be ineffective in curing patients infected with Azith^R^ *S.* Typhi with MIC >32 mg/L. The available clinical evidence supports the view that azithromycin demonstrates a good clinical efficacy for the treatment of Azith^S^ typhoid fever^13^ but fails in Azith^R^ typhoid cases identified in our previous study^7^ and in the ACT-South Asia trial. These findings underscore the strong predictive value and robust correlation of the intracellular killing model with the clinical treatment efficacy of azithromycin. Accordingly, this model serves as an invaluable tool for assessing the efficacy of azithromycin and other antibiotics for typhoid treatment.

We also note that, although azithromycin accumulates to high levels within THP-1 macrophages, its maximal efficacy against intracellular *S.* Typhi did not reach a bactericidal effect. Furthermore, the intracellular static concentrations required for growth inhibition are generally higher than the MIC values determined in extracellular conditions. These findings suggest that additional factors within the intracellular environment may influence azithromycin activity. After being engulfed by macrophage, *S.* Typhi resides within the *Salmonella*-containing vacuole, a modified phagolysosome characterised by an acidic pH ranging from 4.0 to 5.0.^36^ Notably, the antibacterial activity of azithromycin is markedly reduced under such acidic conditions.^37^^,^^38^ This reduced intracellular efficacy may contribute to the delayed bacterial clearance observed clinically, potentially explaining the prolonged fever clearance times seen in typhoid patients treated with azithromycin.^35^

There are limitations to this study that should be acknowledged. First, the THP-1 macrophage model may not fully reflect the behaviour and functionality of primary human monocyte-derived macrophages, nor their complex interactions with human immune cells during infection. Furthermore, the study did not examine the subcellular distribution of azithromycin within infected THP-1 macrophages, which could provide further insights into the drug's intracellular activity. Our pharmacological model assumes a fixed Hill slope and a simplified relationship between extracellular and intracellular azithromycin concentrations. However, these assumptions do not affect the robustness of our intracellular model and data interpretation. Additionally, clinical data on azithromycin treatment response in azithromycin-resistant typhoid cases remains limited. Further evaluation in larger patient cohorts is needed to establish the clinical utility of our model in predicting treatment outcomes.

In conclusion, we successfully established a THP-1 macrophage infection model to assess the intracellular killing activity of azithromycin against both azithromycin-susceptible and azithromycin-resistant *S.* Typhi isolates. Azithromycin showed a dose-dependent killing effect in intracellular conditions, but only a bacteriostatic effect in extracellular conditions. Our intracellular model indicates that the current azithromycin regimen is effective against azithromycin-susceptible *S.* Typhi, but is unlikely to cure infections caused by azithromycin-resistant strains. These findings highlight the urgent need for continued surveillance of azithromycin resistance in typhoid endemic areas and the development of alternative treatment options should resistant strains become widespread.

## Contributors

NNPT conducted the experiments, data analyses, interpreted the results and drafted the manuscript. VVP and NHTT performed the experiments and edited the manuscript. QN, CV, SD, and SB supported the sample and data collection and edited the manuscript. PTD designed and supervised the study, interpreted the results and edited the manuscript. GET, BB, CMP, SB, MAR, JCDH, AK contributed to the data interpretation and editing of the manuscript. All authors read and approved the final draft. NNPT and PTD had full access to the underlying data and verified its accuracy.

## Data sharing statement

The raw Illumina data of the study are deposited in the European Nucleotide Archive (Project Number: PRJEB98584). We confirm the availability of the clinical outcome data from the two ACT-SA trial participants and the raw killing assay/pharmacodynamic data. The corresponding author hold these datasets and will make them available upon reasonable request.

## Declaration of interests

The authors declare no competing interests.

## References

[1] Kalra S.P., Naithani N., Mehta S.R., Swamy A.J. (2003). Current trends in the management of typhoid fever. Med J Armed Forces India.

[2] Wain J., Hendriksen R.S., Mikoleit M.L., Keddy K.H., Ochiai R.L. (2015). The Lancet.

[3] Crump J.A., Sjolund-Karlsson M., Gordon M.A., Parry C.M. (2015). Epidemiology, clinical presentation, laboratory diagnosis, antimicrobial resistance, and antimicrobial management of invasive salmonella infections. Clin Microbiol Rev.

[4] (2023). World Health Organization Model List of Essential Medicines – 23rd List, 2023.

[5] Klemm E.J., Shakoor S., Page A.J. (2018). Emergence of an Extensively Drug-Resistant Salmonella Enterica Serovar Typhi Clone Harboring a Promiscuous Plasmid Encoding Resistance to Fluoroquinolones and Third-Generation Cephalosporins. mBio.

[6] Sah R., Donovan S., Seth-Smith H.M.B. (2020). A novel lineage of ceftriaxone-resistant Salmonella Typhi from India that is closely related to XDR S. Typhi found in Pakistan. Clin Infect Dis.

[7] Duy P.T., Dongol S., Giri A. (2020). The emergence of azithromycin-resistant Salmonella Typhi in Nepal. JAC Antimicrob Resist.

[8] Octavia S., Chew K.L., Lin R.T.P., Teo J.W.P. (2021). Azithromycin-resistant Salmonella enterica serovar typhi AcrB-R717Q/L, Singapore. Emerg Infect Dis.

[9] Carey M.E., Jain R., Yousuf M. (2021). Spontaneous Emergence of Azithromycin Resistance in Independent Lineages of Salmonella Typhi in Northern India. Clin Infect Dis.

[10] Iqbal J., Dehraj I.F., Carey M.E. (2020). A race against time: reduced Azithromycin Susceptibility in Salmonella enterica Serovar Typhi in Pakistan. mSphere.

[11] Hooda Y., Sajib M.S.I.I., Rahman H. (2019). Molecular mechanism of azithromycin resistance among typhoidal Salmonella strains in Bangladesh identified through passive pediatric surveillance. PLoS Negl Trop Dis.

[12] Clinical and Laboratory Standards Institute (2015). Performance standards for antimicrobial susceptibility testing; twenty-fifth informational supplement, M100-S25 edn. https://clsi.org/shop/standards/m100/.

[13] Parry C.M., Thieu N.T.V., Dolecek C. (2015). Clinically and microbiologically derived azithromycin susceptibility breakpoints for Salmonella enterica serovars Typhi and Paratyphi A. Antimicrob Agents Chemother.

[14] Bartold P.M., Du Bois A.H., Gannon S., Haynes D.R., Hirsch R.S. (2013). Antibacterial and immunomodulatory properties of azithromycin treatment implications for periodontitis. Inflammopharmacology.

[15] Matzneller P., Krasniqi S., Kinzig M. (2013). Blood, tissue, and intracellular concentrations of azithromycin during and after end of therapy. Antimicrob Agents Chemother.

[16] Wildfeuer A., Laufen H., Zimmermann T. (1996). Uptake of azithromycin by various cells and its intracellular activity under in vivo conditions. Antimicrob Agents Chemother.

[17] Arjyal A., Basnyat B., Nhan H.T. (2016). Gatifloxacin versus ceftriaxone for uncomplicated enteric fever in Nepal: an open-label, two-centre, randomised controlled trial. Lancet Infect Dis.

[18] Giri A., Karkey A., Dongol S. (2021). Azithromycin and cefixime combination versus azithromycin alone for the out-patient treatment of clinically suspected or confirmed uncomplicated typhoid fever in South Asia: a randomised controlled trial protocol. Wellcome Open Res.

[19] Andrews S. (2010). FastQC: a quality control tool for high throughput sequence data. https://www.bioinformatics.babraham.ac.uk/projects/fastqc/.

[20] Carey M.E., Dyson Z.A., Ingle D.J. (2023). Global diversity and antimicrobial resistance of typhoid fever pathogens: insights from a meta-analysis of 13,000 Salmonella Typhi genomes. Elife.

[21] Tsuchiya S., Yamabe M., Yamaguchi Y., Kobayashi Y., Konno T., Tada K. (1980). Establishment and characterization of a human acute monocytic leukemia cell line (THP-1). Int J Cancer.

[22] Starr T., Bauler T.J., Malik-Kale P., Steele-Mortimer O. (2018). The phorbol 12-myristate-13-acetate differentiation protocol is critical to the interaction of THP-1 macrophages with Salmonella Typhimurium. PLoS One.

[23] Wang J., Ma S., Li W. (2021). Salmonella enterica serovar typhi induces host metabolic reprogramming to increase glucose availability for intracellular replication. Int J Mol Sci.

[24] Forest C.G., Ferraro E., Sabbagh S.C., Daigle F. (2010). Intracellular survival of Salmonella enterica serovar Typhi in human macrophages is independent of Salmonella pathogenicity island (SPI)-2. Microbiology (Reading).

[25] Lathrop S.K., Cooper K.G., Binder K.A. (2018). Salmonella Typhimurium Infection of Human Monocyte-Derived Macrophages. Curr Protoc Microbiol.

[26] Nguyen H.A., Denis O., Vergison A., Tulkens P.M., Struelens M.J., Van Bambeke F. (2009). Intracellular activity of antibiotics in a model of human THP-1 macrophages infected by a Staphylococcus aureus small-colony variant strain isolated from a cystic fibrosis patient: study of antibiotic combinations. Antimicrob Agents Chemother.

[27] Sampson M.R., Dumitrescu T.P., Brouwer K.L.R., Schmith V.D. (2014). Population pharmacokinetics of azithromycin in whole blood, peripheral blood mononuclear cells, and polymorphonuclear cells in healthy adults. CPT Pharmacometrics Syst Pharmacol.

[28] Krombach F., Munzing S., Allmeling A.M., Gerlach J.T., Behr J., Dorger M. (1997). Cell size of alveolar macrophages: an interspecies comparison. Environ Health Perspect.

[29] Mélard A., Garcia L.G., Das D. (2013). Activity of ceftaroline against extracellular (broth) and intracellular (THP-1 monocytes) forms of methicillin-resistant Staphylococcus aureus: Comparison with vancomycin, linezolid and daptomycin. J Antimicrob Chemother.

[30] Liu P., Allaudeen H., Chandra R. (2007). Comparative pharmacokinetics of azithromycin in serum and white blood cells of healthy subjects receiving a single-dose extended-release regimen versus a 3-day immediate-release regimen. Antimicrob Agents Chemother.

[31] World Health Organization (2019). Typhoid vaccines: WHO position paper, March 2018—recommendations. Vaccine.

[32] Nizamuddin S., Khan E., Chattaway M.A., Godbole G. (2023). Case of carbapenem-resistant Salmonella Typhi infection, Pakistan, 2022. Emerg Infect Dis.

[33] Manesh A., Balaji V., Kumar D.R.N., Rupali P. (2017). A case of clinical and microbiological failure of azithromycin therapy in Salmonella enterica serotype Typhi despite low azithromycin MIC. Int J Infect Dis.

[34] Rakita R.M., Jacques-Palaz K., Murray B.E. (1994). Intracellular activity of azithromycin against bacterial enteric pathogens. Antimicrob Agents Chemother.

[35] Jin C., Gibani M.M., Pennington S.H. (2019). Treatment responses to azithromycin and ciprofloxacin in uncomplicated Salmonella Typhi infection: a comparison of clinical and microbiological data from a controlled human infection model. PLoS Negl Trop Dis.

[36] Rathman M., Sjaastad M.D., Falkow S. (1996). Acidification of phagosomes containing Salmonella typhimurium in murine macrophages. Infect Immun.

[37] Butler T., Frenck R.W., Johnson R.B., Khakhria R. (2001). In vitro effects of azithromycin on Salmonella typhi: early inhibition by concentrations less than the MIC and reduction of MIC by alkaline pH and small inocula. J Antimicrob Chemother.

[38] Retsema J.A., Brennan L.A., Girard A.E. (1991). Effects of environmental factors on the in vitro potency of azithromycin. Eur J Clin Microbiol Infect Dis.

