# Supplementary material for: Determining intracellular and extracellular activities of azithromycin against azithromycin-resistant Salmonella Typhi and their association with clinical treatment responses
Source: eBioMedicine. 2026 Aug 22;131:106447. doi: 10.1016/j.ebiom.2026.106447 (PMC13524848; doi:10.1016/j.ebiom.2026.106447)
Supplement: Supplementary file 1 [file mmc1.docx]

**Supplementary Table 1**. Metadata of isolates used in this study

| Isolates | Location | Year | Reference | Azithromycin MIC (mg/L) | AcrB mutation | Genotype | Accession number |
| --- | --- | --- | --- | --- | --- | --- | --- |
| 02TY186 | Nepal | 2013 | PMID: 26809813 | 6 | None | 4.3.1.2 | ERR1079288 |
| 02TY216 | Nepal | 2014 | PMID: 26809813 | 6 | None | 4.3.1.2 | ERR1079296 |
| 02TY027 | Nepal | 2012 | PMID: 26809813 | 8 | None | 4.3.1.2 | ERR1079237 |
| 02TY176 | Nepal | 2013 | PMID: 26809813 | 8 | None | 4.3.1.2 | ERR1079284 |
| 02TY150 | Nepal | 2013 | PMID: 26809813 | 12 | None | 4.3.1.2 | ERR1079273 |
| 02TY173 | Nepal | 2013 | PMID: 26809813 | 12 | None | 4.3.1.2 | ERR1079282 |
| 02TY015 | Nepal | 2012 | PMID: 26809813 | 16 | None | 4.3.1.2 | ERR1079234 |
| 02TY135 | Nepal | 2013 | PMID: 26809813 | 16 | None | 4.3.1.2 | ERR1079264 |
| ST1997 | Nepal | 2019 | PMID: 34223059 | >256 | R717L | 4.3.1 | ERR4042542 |
| ST2004 | Nepal | 2019 | PMID: 34223059 | >256 | R717L | 4.3.1 | ERR4042543 |
| ST2019 | Nepal | 2020 | PMID: 34223059 | >256 | R717L | 4.3.1 | ERR4042544 |
| ST2098 | Nepal | 2020 | This study | 128 | R717L | 4.3.1 | ERR15665756 |
| ST2113 | Nepal | 2020 | This study | 128 | R717L | 4.3.1 | ERR15665757 |
| ST2124 | Nepal | 2020 | This study | 96 | R717L | 4.3.1.2 | ERR15665758 |
| ACT105-032 | Nepal | 2023 | This study | >256 | R717Q | 4.3.1.2 | ERR15665754 |
| ACT105-033 | Nepal | 2023 | This study | >256 | R717Q | 4.3.1.2 | ERR15665755 |

**Supplementary Table 2**. Pharmacological parameters estimated from dose-response sigmoidal model of azithromycin against each Azith^S^ *S*. Typhi isolate.

| ***S*. *Typhi* isolates** | **MIC**  **(mg/L)** | **E_min_ (95% CI) ^a^** | **E_max_ (95% CI) ^b^** | **EC_50_ ^c^**  **(xMIC)** | **EC_50i_ ^d^**  **(mg/L)** | **C_s_ ^e^**  **(xMIC)** | **C_si_ ^f^**  **(mg/L)** | **R^2^** | **Adjusted R^2^** |
| --- | --- | --- | --- | --- | --- | --- | --- | --- | --- |
| Intracellular |  |  |  |  |  |  |  |  |  |
| 02TY186 | 6 | 0.31 (0.19 to 0.44) | -2.96 (-4.49 to -2.25) | 3.35 | 396.75 | 0.63 | 75.13 | 0.972 | 0.968 |
| 02TY216 | 6 | 0.97 (0.58 to 1.80) | -0.75 (-0.98 to -0.55) | 0.39 | 46.89 | 0.50 | 59.65 | 0.881 | 0.865 |
| 02TY027 | 8 | 1.56 (1.19 to 2.04) | -1.72 (-1.94 to -1.52) | 0.47 | 74.28 | 0.42 | 66.82 | 0.971 | 0.968 |
| 02TY176 | 8 | 0.36 (0.16 to 0.58) | -1.26 (-1.46 to -1.10) | 0.88 | 139.05 | 0.25 | 39.79 | 0.940 | 0.932 |
| 02TY150 | 12 | 0.60 (0.39 to 0.82) | -1.78 (-2.08 to -1.54) | 1.31 | 310.98 | 0.43 | 102.61 | 0.960 | 0.955 |
| 02TY173 | 12 | 0.59 (0.36 to 0.85) | -1.83 (-2.03 to -1.66) | 0.74 | 176.21 | 0.23 | 54.91 | 0.969 | 0.965 |
| 02TY015 | 16 | 0.59 (0.35 to 0.85) | -1.96 (-2.25 to -1.72) | 1.05 | 334.84 | 0.31 | 98.66 | 0.960 | 0.954 |
| 02TY135 | 16 | 0.49 (0.32 to 0.67) | -2.07 (-2.39 to -1.81) | 1.77 | 561.95 | 0.41 | 130.45 | 0.973 | 0.969 |
| Extracellular |  |  |  |  |  |  |  |  |  |
| 02TY186 | 6 | 3.57 (3.17 to 3.99) | -0.85 (-1.24 to -0.49) | 0.21 | NA | 0.89 | NA | 0.920 | 0.904 |
| 02TY216 | 6 | 3.57 (3.32 to 3.84) | -0.49 (-0.71 to -0.28) | 0.18 | NA | 1.32 | NA | 0.961 | 0.958 |
| 02TY027 | 8 | 3.78 (3.51 to 4.05) | -0.66 (-0.85 to -0.47) | 0.13 | NA | 0.74 | NA | 0.968 | 0.966 |
| 02TY176 | 8 | 3.86 (3.42 to 4.32) | -0.71 (-1.06 to -0.38) | 0.14 | NA | 0.78 | NA | 0.917 | 0.912 |
| 02TY150 | 12 | 3.88 (3.60 to 4.18) | -0.98 (-1.27 to -0.72) | 0.24 | NA | 0.91 | NA | 0.961 | 0.959 |
| 02TY173 | 12 | 4.08 (3.74 to 4.43) | -1.07 (-1.40 to -0.76) | 0.22 | NA | 0.85 | NA | 0.951 | 0.948 |
| 02TY015 | 16 | 3.92 (3.62 to 4.23) | -1.62 (-1.90 to -1.35) | 0.21 | NA | 0.49 | NA | 0.969 | 0.967 |
| 02TY135 | 16 | 4.03 (3.45 to 4.64) | -1.27 (-1.75 to -0.83) | 0.15 | NA | 0.49 | NA | 0.891 | 0.884 |

^a^ Minimal relative efficacy (in log_10_CFU/mL, 95% confidence interval): The increase in colony-forming units (CFU) for an infinitely low antibiotic concentration compared to the initial inoculum.

^b^ Maximal relative efficacy (in log_10_CFU/mL, 95% confidence interval): The decrease in CFU for an infinitely high antibiotic concentration compared to the initial inoculum.

^c^ Extracellular concentration of azithromycin (in multiples of MIC) causing a reduction halfway between E_min_ and E_max_, as obtained from the Hill equation (with slope factor of 1).

^d^ Intracellular concentration of azithromycin (in mg/L) causing a reduction halfway between E_min_ and E_max_, as calculated from ^c^ and equation of the non-linear curve showing the accumulation of azithromycin into THP-1 macrophage after 24-hour incubation (figure 1D).

^e^ Extracellular concentration of azithromycin (in multiples of MIC) resulting in no apparent bacterial growth compared to the initial inoculum, as determined by graphical interpolation.

^f^ Intracellular concentration of azithromycin (in mg/L) resulting in no apparent bacterial growth compared to the initial inoculum, as calculated from e and equation of the non-linear curve showing the accumulation of azithromycin into THP-1 macrophage after 24-hour incubation (figure 1D).

NA: not applicable

**Supplementary Table 3.** Results from Kruskal-Wallis test comparison of fluorescence levels in the PI uptake assay THP-1 macrophage interact with *S.* Typhi

| **Comparison** | **Mean rank difference** | **Summary** | **P-value** |
| --- | --- | --- | --- |
| Uninfected THP-1 vs. 100% cell death | -11.00 | * | 0.030 |
| Uninfected THP-1 vs. THP-1 + 02TY150 | -0.6667 | ns | 0.895 |
| Uninfected THP-1 vs. THP-1 + 02TY173 | -4.333 | ns | 0.392 |
| Uninfected THP-1 vs. THP-1 + 02TY216 | -4.000 | ns | 0.429 |

**Supplementary Table 4.** Results from Kruskal-Wallis test comparison of fluorescence levels in the PI uptake assay for THP-1 macrophages exposed to azithromycin for 24 h

| **Comparison** | **Mean rank difference** | **Summary** | **P-value** |
| --- | --- | --- | --- |
| Negative control vs. 6 mg/L | -1.000 | ns | 0.926 |
| Negative control vs. 8 mg/L | -3.333 | ns | 0.756 |
| Negative control vs. 16 mg/L | -10.67 | ns | 0.320 |
| Negative control vs. 32 mg/L | -7.333 | ns | 0.494 |
| Negative control vs. 64 mg/L | -14.33 | ns | 0.181 |
| Negative control vs. 96 mg/L | -18.00 | ns | 0.093 |
| Negative control vs. 128 mg/L | -5.667 | ns | 0.597 |
| Negative control vs. 150 mg/L | -21.33 | * | 0.047 |
| Negative control vs. 170 mg/L | -26.00 | * | 0.015 |
| Negative control vs. 180 mg/L | -24.67 | * | 0.021 |
| Negative control vs. 192 mg/L | -29.67 | ** | 0.006 |
| Negative control vs. 256 mg/L | -33.00 | ** | 0.002 |

**Supplementary Figure 1. Killing effects of azithromycin against intracellular and extracellular Azith^S^ *S*. Typhi at different concentrations.
Left panels:** Intracellular activity of azithromycin against eight Azith^S^ isolates in THP-1 macrophages over 24 h. **Right panels:** Extracellular activity of azithromycin against the same isolates over 24 h. Each graph illustrates the time- and dose-killing curves, represented as changes in CFU per mL (Δ log_10_CFU/mL; means ± SDs; n = 3; most SD bars are smaller than the symbols) from the initial inoculum.

**Supplementary figure 2. Killing effects of azithromycin against intracellular and extracellular Azith^R^ *S*. Typhi at different concentrations.
Left panels:** Intracellular activity of azithromycin against eight Azith^R^ isolates in THP-1 macrophages over 24h. **Right panels:** Extracellular activity of azithromycin against the same isolates over 24 h. Each graph illustrates the time- and dose-killing curves, represented as changes in CFU per mL (Δ log_10_CFU/mL; means ± SDs; n = 3; most SD bars are smaller than the symbols) from the initial inoculum.
